# Loss of the Y Chromosome in Oral Potentially Premalignant Disorders Predicts Malignant Progression: An Integrative Cross‐Species Multi‐Cohort Bioinformatic Study

**DOI:** 10.1002/hed.70070

**Published:** 2025-10-22

**Authors:** Rui Han, Jos B. Poell, Cristina Conde‐Lopez, Tuula Salo, Ina Kurth, Ruud H. Brakenhoff, Jochen Hess

**Affiliations:** ^1^ Department of Otorhinolaryngology, Head and Neck Surgery Heidelberg University Hospital Heidelberg Germany; ^2^ Amsterdam UMC, Vrije Universiteit Amsterdam Otolaryngology ‐ Head and Neck Surgery Amsterdam the Netherlands; ^3^ Cancer Center Amsterdam Cancer Biology and Immunology Amsterdam the Netherlands; ^4^ German Cancer Research Center (DKFZ) Heidelberg, Division of Radiooncology Radiobiology Germany; ^5^ Department of Oral and Maxillofacial Diseases, Clinicum, Faculty of Medicine University of Helsinki, Helsinki University Hospital Helsinki Finland; ^6^ Translational Immunology Research Program (TRIMM) University of Helsinki Helsinki Finland; ^7^ iCAN Digital Precision Cancer Medicine Flagship University of Helsinki Helsinki Finland; ^8^ Oral Pathology, Research Unit of Population Health, Faculty of Medicine University of Oulu Oulu Finland; ^9^ Medical Research Center, Oulu University Hospital University of Oulu Oulu Finland; ^10^ German Cancer Research Center (DKFZ), and German Cancer Consortium (DKTK), Core Center Heidelberg Heidelberg Germany; ^11^ National Center for Radiation Research in Oncology (NCRO) Heidelberg Institute for Radiation Oncology (HIRO) Heidelberg Germany

**Keywords:** extreme down‐regulation of Y chromosome gene expression (EDY), loss of the Y chromosome (LOY), lysine demethylase 5D (KDM5D), oral potentially premalignant disorders (OPMD), oral squamous cell carcinoma (OSCC)

## Abstract

**Background:**

The loss of the Y chromosome (LOY) and the extreme down‐regulation of Y chromosome gene expression (EDY) are frequently observed in oral squamous cell carcinoma (OSCC). However, their roles in oral potentially malignant disorders (OPMDs) are unclear.

**Methods:**

A comprehensive bioinformatic analysis was performed using publicly available datasets from chemically induced mouse OSCC models and human cohorts. The analysis included LOY/EDY detection, gene set variation analysis (GSVA), PROGENy pathway profiling, cell‐to‐cell communication inference, and epigenetic correlation studies.

**Results:**

LOY was prevalent among men with OPMD, and EDY was identified in both mouse models and human OPMDs. The presence of LOY/EDY was associated with a higher risk of OPMD progression to OSCC. Single‐cell analysis revealed that EDY‐positive epithelial cells exhibited elevated oncogenic pathway activity and enhanced IL17‐IL17RC signaling, possibly due to the loss of KDM5D in epithelial cells and altered epigenetic regulation.

**Conclusions:**

LOY/EDY can be detected in OPMD and promotes malignant progression by altering oncogenic signaling and epithelial cell interactions. LOY/EDY may serve as both a diagnostic biomarker and a therapeutic target, improving clinical management and patient outcomes.

## 1 Introduction

Loss of the Y chromosome (LOY) is a significant genomic event in aging and age‐related diseases characterized by the partial or complete absence of the Y chromosome from the cell's genome [1], with clinically relevant implications that vary by disease. This process is accompanied by the reduction in the expression of genes located on the Y chromosome, which is known as extreme downregulation of Y chromosome gene expression (EDY) [1]. Studies have confirmed a high correlation between EDY and LOY, indicating that EDY is likely a functional consequence of LOY [1].

As men age, the incidence of LOY in normal cells, in particular hematopoietic cells, increases, suggesting that LOY is a common age‐related phenomenon [2]. This increase in LOY is linked to various age‐related diseases [3], including Alzheimer's disease [4], macular degeneration [5], and heart attack, among others [6]. LOY may contribute to the pathogenesis of these conditions, potentially through mechanisms related to genomic instability and cell cycle regulation [7].

LOY has been identified as a common somatic event associated with an increased risk of all‐cause mortality and non‐hematological cancer mortality [8]. This association is evident across various tumor types in which LOY prevalence exceeds that of certain well‐known cancer driver genes [9]. In the cancer context, LOY is notably enriched in tumors with genomic instability, which correlates with broader genomic features beyond the Y chromosome [10], including TP53 mutations and oncogene amplifications such as KRAS and EGFR, and may contribute to the higher incidence rates of cancer in men.

In bladder cancer, LOY‐associated tumors promote CD8^+^ T cell exhaustion within the tumor microenvironment, rendering them more responsive to immune checkpoint blockade therapies [11]. This finding points to a functional consequence of LOY that might influence therapeutic strategies in oncological treatment.

In the past, most research on LOY and EDY has centered on implications for cancer susceptibility, cancer prognosis, and treatment response. However, while LOY has been implicated as a potential driver in head and neck squamous cell carcinoma (HNSCC) [9], its role during the early steps of tumorigenesis, particularly in premalignant stages, remains largely unexplored. Oral squamous cell carcinoma (OSCC) is the most common form of HNSCC diagnosed, and similar to other solid tumors, emerges through a complex multistep process. OSCC may arise in the squamous epithelium from potentially malignant disorders (OPMDs) or de novo, followed by pro‐tumorigenic mutations. OPMDs, which include different subtypes [12], significantly heighten the risk of developing OSCC. However, not all OPMD cases have the same risk to progress to OSCC, suggesting variability in the molecular pathways involved [13].

Histologically, some OPMDs are characterized by features such as hyperplasia, hyperkeratosis, and various levels of dysplasia [14]. The molecular changes driving the transition from normal oral tissue to OPMD and OSCC are still under investigation. Understanding these changes is essential for advancing early diagnosis, risk assessment, and targeted therapies for both conditions.

By examining EDY and LOY across normal tissues, different OPMD subtypes, and OSCC, the potential relationship between EDY/LOY and OSCC development was investigated. Notably, EDY and LOY, despite their potential clinical relevance, have not been analyzed in the 4‐NQO mouse models. The main objective of this study is the identification of molecular biomarkers as risk factors for malignant progression into OSCC, which may enhance personalized treatment for OPMD and could lead to the development of targeted therapies.

Importantly, this study was exploratory in nature. Rather than aiming to establish definitive causality, we sought to identify biologically plausible trends and generate hypotheses regarding whether EDY/LOY might serve as early, male‐specific risk markers for malignant progression in OPMD. These findings provide a solid basis for future validation in larger, multicenter cohorts.

## 2 Materials and Methods

### 2.1 Data Collection

In this study, publicly available omics data were used to analyze EDY and LOY in both 4‐NQO mouse models and human patients. All the datasets utilized in this study were downloaded from the Gene Expression Omnibus (GEO) and CodeOcean (https://codeocean.com/capsule/0664927/tree/v1). These datasets include samples from both human and mouse origins, a full summary of each dataset—including accession numbers, data types, the number of male samples, and platform—is provided in Table S1. No further inclusion or exclusion criteria were applied beyond selecting samples classified as normal mucosa, OPMD, or HNSCC. No a priori sample size calculation was performed, as the study was retrospective and relied on publicly available data. The primary clinical endpoint examined was malignant progression from OPMD to cancer, which follows the original study. Among the datasets used, only GSE26549 and the Amsterdam UMC cohort contained longitudinal follow‐up information suitable for this analysis. Other datasets did not include clinical endpoints and were therefore not used to evaluate progression‐related endpoints. The 4‐NQO mouse model is a well‐established tool for studying the multistep process of oral carcinogenesis, mirroring human OSCC development and enabling analysis of histological and molecular changes from normal mucosa to invasive carcinoma [15]. Key elements of this model include its ability to replicate human disease stages and genetic mutations related to oral cancer progression [16], making it valuable for studying early steps in oral carcinogenesis.

### 2.2 Processing of Amsterdam UMC Cohort

The genomic data analyzed were derived from a retrospective cohort of patients with oral leukoplakia, as reported by Wils et al. [17]. The original study investigated the presence of genomic copy number alterations in a cohort of 89 patients, including 28 men.

The data were segmented using the segmentBins function from QDNAseq [18], and missing values were removed from Y chromosome segments. The mean copy number of the Y chromosome, referred to as the Y chromosme signal, was calculated and then normalized for cellularity and corrected for ploidy, resulting in the adjusted Y chromosome signal (Figure S2C,D and Figure 2F).

### 2.3 Processing of scRNA‐Seq Data

The raw data of the gene expression matrix was downloaded from GEO and was filtered using the “Seurat” R package (5.0.1) [19]. Only cells with expression of > 200 genes and < 8000 genes and < 10% of mitochondrial gene expression in UMI counts, and genes with expression in > 0.1% cells were considered for further analysis. Cells were annotated according to the original publications.

Genes on the Y chromosome with more than 0.05 average normalized expression and/or with expression in > 5% of cells were considered. Cells were classified as EDY if no expression of these genes was detected using UMI counts [20]. Dataset‐specific detection sensitivity may alter the final Y chromosome‐gene list even when identical thresholds are applied. For GSE181919, EDY classification was based on RPS4Y1, TTTY15, DDX3Y, and EIF1AY transcript levels.

Epithelial cells from women's samples served as a reference and showed no expression of Y chromosome genes, demonstrating the robustness and quality of the data (Figure S3A). Additional filters were applied to limit variability and technical biases. Only minor differences were detected in the percentage of mitochondrial and ribosomal genes, the number of genes, and total transcript counts between the EDY and non‐EDY epithelial cells (Figure S3B–E).

The expression of specific gene sets in cells was analyzed using the “AddModuleScore” function in Seurat.

### 2.4 PROGENy Pathway Analysis

DecoupleR (2.9.7) [21] is a package that integrates various statistical methods to extract biological signatures from prior knowledge, accounting for the sign and weight of network interactions. PROGENy provides a curated set of pathways and associated target genes, each assigned interaction weights. Following the recommended workflow in the official documentation, the normalized, log‐transformed count matrix was used as input. In this study, the multivariate linear model (MLM) was applied to detect biological activity from gene expression data. A positive score indicates pathway activation, while a negative score suggests reduced or inactive pathway activity.

### 2.5 Cell‐to‐Cell Interaction Analysis

CellChat software (1.4.0) [22, 23] was utilized to infer and visualize the communication between various cell subsets using the GSE181919 dataset. The analysis of ligand‐receptor interactions was performed using a Seurat normalized expression matrix. The known ligand‐receptor pairs were downloaded from CellChatDB, a database backed by literature that includes documented interactions between ligands and receptors in both mice and humans.

### 2.6 Gene Set Variation Analysis

Gene Set Variation Analysis (GSVA) was performed using the GSVA package (1.50.0) [24]. In the case of human RNA‐seq data, the following Y chromosome genes were used: DDX3Y, KDM5D, ZFY, UTY, USP9Y, EIF1AY, RPS4Y1, PRKY, NLGN4Y, TMSB4Y, SRY, and TBL1Y. For mouse RNA‐seq data, the analysis includes all protein‐coding genes that are encoded on the Y chromosome.

### 2.7 Statistical Analysis

Standard non‐parametric tests were employed for the statistical analysis of the data. Specifically, the Wilcoxon rank‐sum test was used for comparisons between two independent groups. When data met the assumptions of normality and homogeneity of variance, the Student's *t*‐test was applied. Fisher's exact test was used for analyzing categorical data, with odds ratios (ORs) and corresponding 95% confidence intervals (CIs) calculated to estimate the strength of associations. For ordinal comparisons across ordered tissue groups, the Jonckheere‐Terpstra trend test was utilized to assess monotonic trends. Effect sizes were quantified using Cohen's d to interpret the magnitude of group differences. For the dataset GSE164619, permutation tests (10 000 resamples) were conducted to improve robustness in small samples. For data visualization, the ggplot2 [25] (3.5.0) and dittoSeq [26] (1.14.2) package were used to generate comprehensive graphics and the pheatmap package (R package version 1.0.12. https://CRAN.R‐project.org/package=pheatmap) for creating heatmaps. All statistical analyses were conducted using R software (4.3.0). The software and packages used for the analyses are detailed in Table S2.

## 3 Results

### 3.1 EDY in the 4‐NQO Induced Mouse Model of OSCC

**FIGURE 1:**
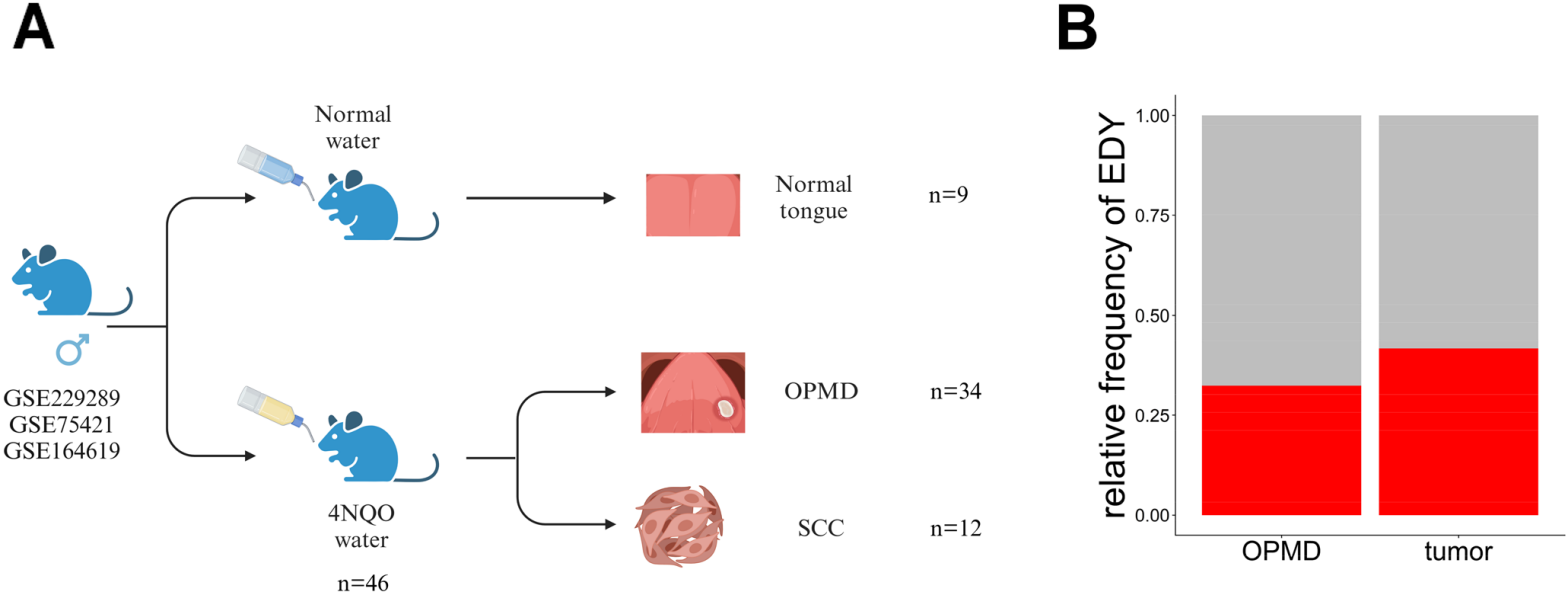
Workflow and analysis of EDY in the 4‐NQO mouse model. (A) Schematic illustration of the mouse models and the available samples with transcriptome data. Created in BioRender. Han, R. (2024) BioRender.com/b95h041. (B) Stacked bar chart showing the percentage of EDY in premalignant (OPMD) and tumor samples of the 4‐NQO mouse model, where gray represents non‐EDY samples and red denotes the EDY group. [Color figure can be viewed at wileyonlinelibrary.com]

To address the question of whether EDY is detectable in the chemically induced mouse model of OSCC and is already present in OPMD, three independent studies were selected: GSE229289 [27], GSE75421 [28], and GSE164619 [29]. These studies provide transcriptome data for different stages of oral tongue carcinogenesis including normal tissue, OPMD, and SCC samples (Figure 1A). Transcript levels for protein‐coding genes of the Y chromosome were assessed for each individual GEO dataset using GSVA, with the respective control‐treated tongues serving as a reference. A sample was classified as EDY if its GSVA score was lower than the minimum score observed in control‐treated tongues. In total, 11 out of 34 OPMD samples (32%) and 5 out of 12 SCC samples (42%) were classified as EDY across the three datasets (Figures 1B and S1). The difference between OPMD and SCC was not statistically significant (OR = 1.49, 95% CI: 0.39–5.78, *p* = 0.73; Fisher's exact test), suggesting that EDY is a consistent feature of chemically induced oral carcinogenesis that is already detectable at the OPMD stage and persists into invasive disease. We next assessed whether EDY/LOY is also evident in OPMD patients and correlates with the risk of malignant transformation.

### 3.2 EDY in OPMD and OSCC From Patients

**FIGURE 2:**
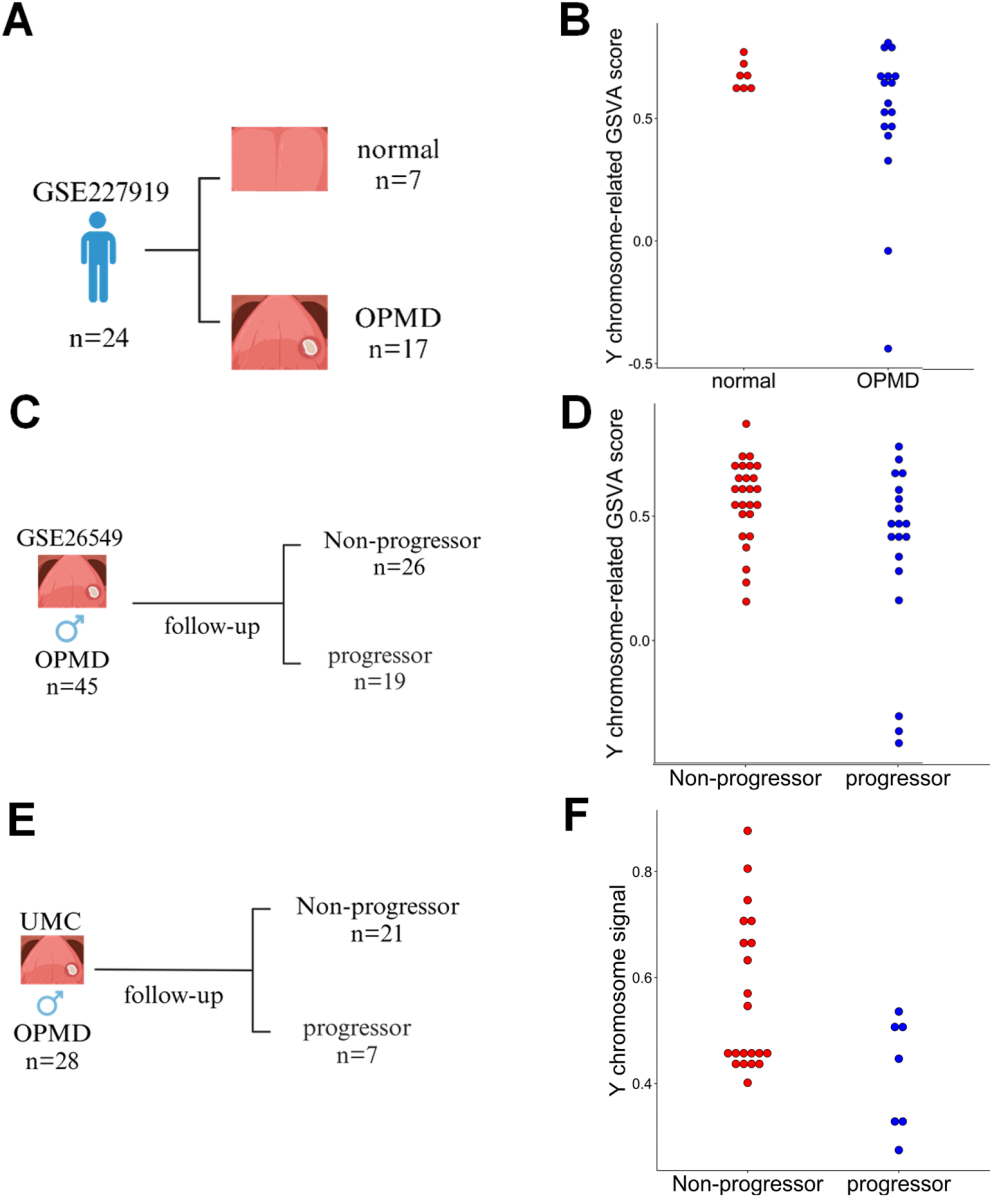
Workflow and analysis of EDY and LOY in human cohorts. Schematic illustration of the samples with available transcriptome (A, C) or genome data (E) from males with OPMD and normal tissue of the oral cavity (A) or OPMD of the oral cavity (C, E). Created in BioRender. Han, R. (2024). (A) BioRender.com/m24o445; (C) BioRender.com/c84s546; (E) BioRender.com/w80k684. Dot plots show the Y chromosome‐related GSVA score for normal and OPMD tissue of males from the GSE227919 dataset (B), and OPMD samples with or without malignant progression to OSCC of males from the GSE26549 dataset (D). (F) Dot plot shows the Y chromosome signal for OPMD samples with or without progressor into OSCC of males from the Amsterdam UMC cohort. [Color figure can be viewed at wileyonlinelibrary.com]

The insights gained from the chemically‐induced mouse model prompted further investigation whether EDY is also detectable in OPMDs from men. A recent study has investigated gene expression changes in premalignant lesions versus normal tissue and OSCC by bulk RNA‐seq [30]. The cohort consists of both men and women, and samples from women were used to assess the quality of the dataset and to determine the appropriate thresholds for further analysis (GSE227919, Figure 2A). As expected, GSVA scores separated most normal mucosa and OPMD samples from men and women (Figure S2A) with a mean GSVA score for male = 0.551 and female = −0.360 (Wilcoxon *p* = 1.4 × 10^−8^). Samples that had a GSVA score below 0.3, representing the highest score observed in women excluding four outliers, were classified as EDY. This classification identified two out of 24 samples (8.3%) from men with EDY (Figure 2B) and raised the question of whether it might serve as a risk factor for malignant progression to OSCC.

To support this hypothesis, bulk RNA‐seq data from an OPMD cohort with clinical follow‐up data were analyzed [31], using samples from women as a reference as described before (GSE26549, Figure 2C). Three OPMD samples that had a GSVA score below 0 were classified as EDY (Figure S2B). In this cohort, all men with OPMD who did not develop OSCC during the follow‐up period were categorized as non‐progressors, whereas those with tumor progression into OSCC were categorized as progressors. All three OPMD patients with EDY (100%) were in the progressor group, whereas 16 out of 42 cases without EDY (38.1%) showed progression into OSCC (Figure 2D). Although the association did not reach statistical significance (Fisher's exact *p* = 0.068, OR = ∞, 95% CI = [0.59–Inf]), these data indicate a potential link between EDY and an increased risk for malignant progression.

### 3.3 LOY in Men With OPMD

As EDY is mainly a consequence of LOY, an OPMD cohort for which genomic and clinical follow‐up data were available was analyzed (Amsterdam UMC cohort, Figure 2E). Among male OPMD patients, those who developed OSCC (*n* = 3) exhibited the lowest Y chromosome scores (indicative of EDY due to LOY) in the cohort. In contrast, patients with the highest Y chromosome scores (*n* = 3, indicative of preserved Y chromosome gene expression) showed no evidence of malignant progression during follow‐up (Figure 2F). This inverse pattern suggests a potential association between reduced Y chromosome expression due to LOY and malignant transformation in a subset of patients. Among 28 male OPMD cases, all three LOY samples progressed to OSCC, compared to only 4 out of 25 non‐LOY cases (Fisher's exact *p* = 0.015; OR = 39; 95% CI = [2.1–∞]). These findings further supported the assumption that EDY derived from LOY serves as a prognostic risk factor for malignant progression. Notably, some male patients with OPMD who progressed to OSCC did not exhibit a marked loss of Y‐linked gene expression, indicating that Y chromosome alteration is not the sole determinant of malignant progression in males, but may contribute in a subset characterized by early EDY/LOY.

### 3.4 EDY in Malignant Epithelial Cells Based on scRNA‐Seq Data Analysis

**FIGURE 3:**
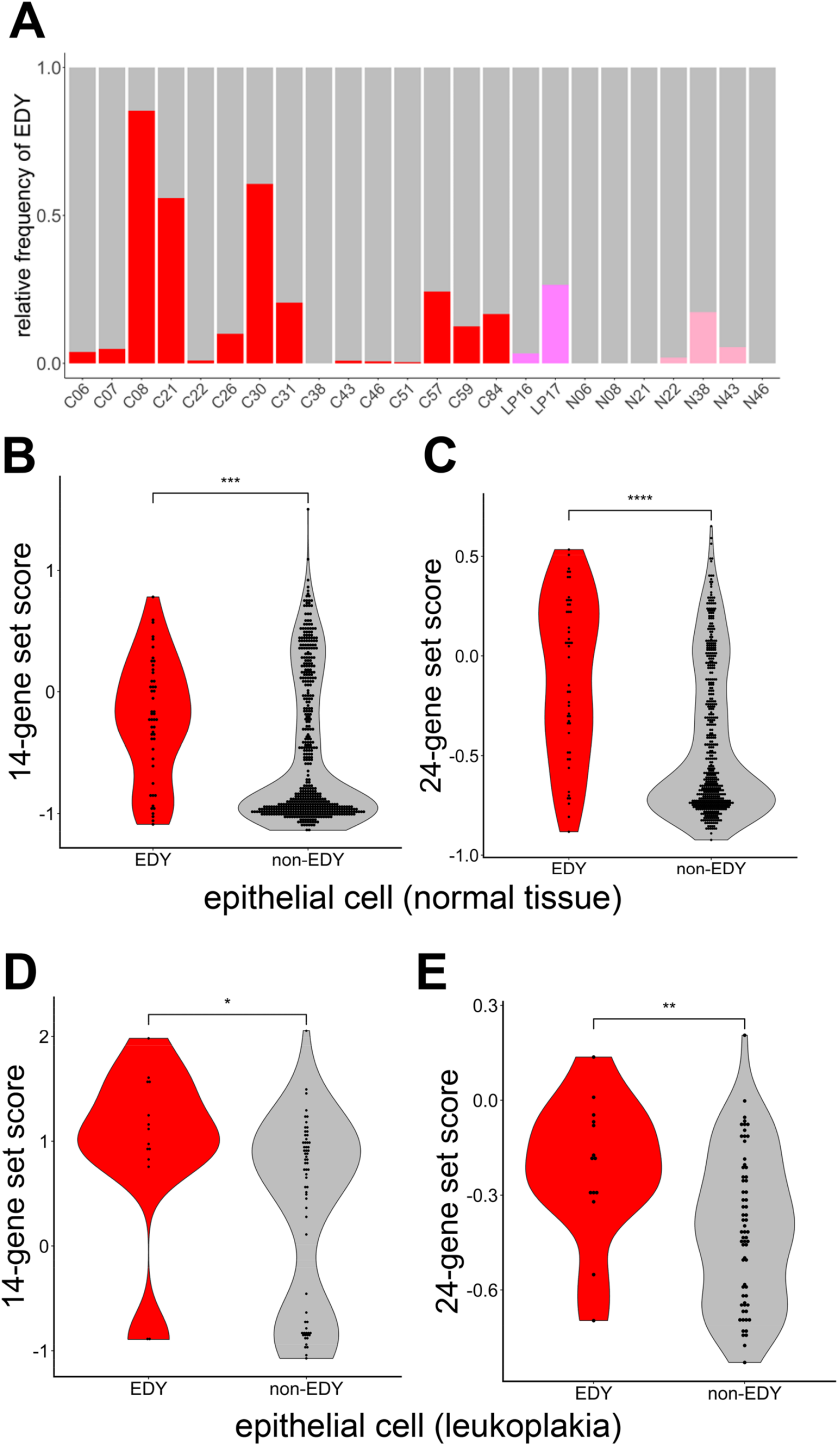
Epithelial cells with EDY exhibit a gene expression pattern related to malignant progression. (A) Bar plot shows the relative frequency of epithelial cells with EDY in normal tissue, leukoplakia, and cancer samples of males from the GSE181919 dataset. Violin plots show the GSVA score based on the 14‐gene set for epithelial cells from normal tissue (B) and leukoplakia (D), and based on the 24‐gene set for epithelial cells from normal tissue (C) and leukoplakia (E) of males from the GSE181919 dataset. *p ≤ 0.05, **p ≤ 0.01, ***p ≤ 0.001, and ****p < 0.0001. [Color figure can be viewed at wileyonlinelibrary.com]

Given the apparent role of Y chromosome gene expression in the early steps of carcinogenesis, the cellular heterogeneity of EDY in potentially premalignant lesions was analyzed using single‐cell RNA sequencing (scRNA‐seq) data of GSE181919 [13]. This dataset includes samples from normal tissue, leukoplakia, and HNSCC. Epithelial cells with EDY were detected in both leukoplakia and almost all tumor samples, but with highly variable proportions (Figure 3A). Notably, epithelial cells with EDY were also detected in three out of seven histologically normal tissues (42.9%), both leukoplakia samples (100%), and 14 out of 15 cancer tissues (93.3%) (Figure 3A). The average proportion of EDY+ epithelial cells increased with histological severity, from 3.5% in normal tissues to 14.9% in leukoplakia and 19.8% in cancers (range: 0.0%–17.3%, 3.3%–26.5%, and 0.0%–85.4%, respectively). This difference in EDY cell proportion across the three tissue groups was statistically significant (Jonckheere–Terpstra trend test, one‐sided *p* = 0.033).

To address the question of whether epithelial cells with EDY share molecular traits of malignant progression, the expression of two previously reported gene sets was analyzed: the canonical marker 14‐gene set (KRT14, KRT17, KRT6A, KRT5, KRT19, KRT8, KRT16, KRT18, KRT6B, KRT15, KRT6C, KRTCAP3, EPCAM, SFN) for malignant cells, and a 24‐gene set (CXCL1, EFNA1, TNFSF10, TM4SF1, KRT19, ELF3, KRT13, CCL20, CCND1, KRT18, CDKN2B, SFN, CDKN2A, NDRG1, SERPINB5, KRT17, KRT8, LAD1, SLC2A1, PGK1, PKP1, TGFBI, NEDD4L, GPC1) differentially expressed in the epithelial cells from histologically normal tissue, clinically leukoplakia, carcinoma in situ, and cancer [13]. As expected, all candidates of the two gene sets exhibited a gradual increase in transcript levels for epithelial cells from normal tissue, leukoplakia, to cancer (Figure S4A–D). The average expression scores were calculated for both gene sets and demonstrated a significantly higher expression in epithelial cells with EDY as compared to the non‐EDY counterparts for normal tissue, leukoplakia, and cancer, except for the 24‐gene set in cancer (Wilcoxon test *p* < 0.05) (Figures 3B–E and S4E,F). However, effect sizes were medium to large in normal tissue (Cohen's *d* = 0.51, 0.91) and leukoplakia (Cohen's *d* = 0.67, 0.82), but negligible in cancer (Cohen's *d* = 0.15, −0.13), suggesting that the EDY status exerts its strongest impact on the transcription of gene sets related to malignant progression in early‐stage lesions.

In summary, this difference in malignant gene expression indicated a more aggressive phenotype of EDY+ epithelial cells, which might explain the elevated risk for malignant progression.

### 3.5 Impact of EDY on Oncogenic Pathway Activity

**FIGURE 4:**
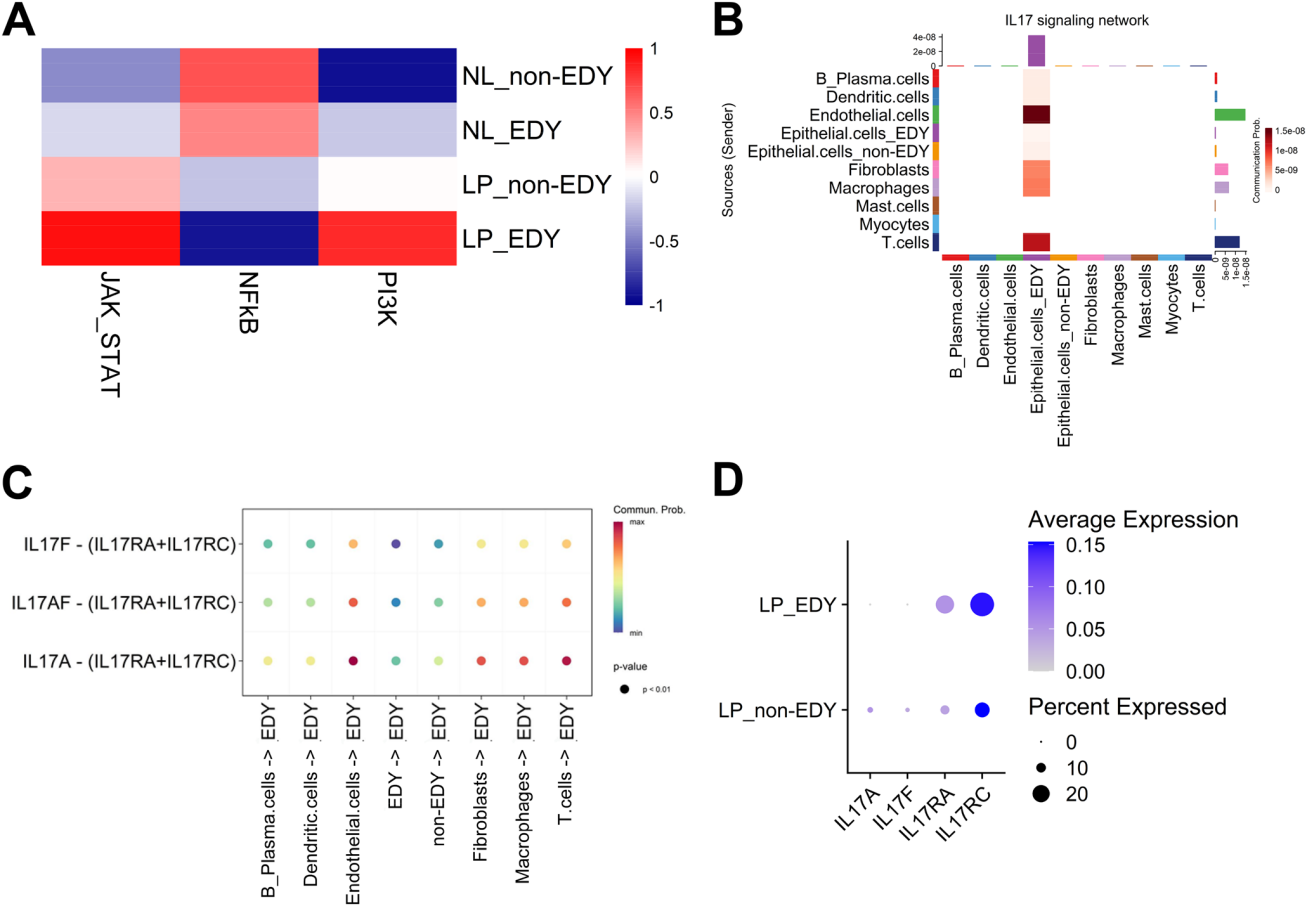
Pathway activity, cell‐to‐cell communication, and IL17 signaling in epithelial cells. (A) Heatmap shows the activity levels of PI3K, JAK–STAT, and NF‐κB pathways in epithelial cells across different pathological conditions, comparing EDY and non‐EDY cells. (B) Heatmap shows cell–cell communication within the IL17 signaling pathway in LP. (C) Bubble plot shows all significant ligand‐receptor (L–R) interactions within the IL17 signaling pathway in LP. Communication probability is represented by color intensity, with higher probabilities in red, circle size reflects statistical significance, with larger circles indicating lower *p*‐values. (D) Bubble plot shows the expression levels of IL17A, IL17F, IL17RA, and IL17RC in leukoplakia and normal tissues epithelial cell. [Color figure can be viewed at wileyonlinelibrary.com]

Differences in oncogenic pathway activities between epithelial cells with or without EDY from normal tissue, leukoplakia, and cancer of the GSE181919 dataset were analyzed using PROGENy (Figure S5). This analysis revealed a significantly higher pathway activity for PI3K (*p* = 0.035) and JAK–STAT signaling (*p* = 0.013) for epithelial cells from leukoplakia with EDY as compared to non‐EDY counterparts, whereas NFkB signaling (*p* = 0.016) was reduced (Figure 4A). A significantly higher pathway activity for PI3K and JAK–STAT signaling (*p* < 0.001) was also evident for EDY+ epithelial cells from normal tissue (Figure 4A), indicating a causal relationship between both oncogenic pathways and Y chromosome gene expression.

### 3.6 Impact of EDY on Cellular Communication

**FIGURE 5:**
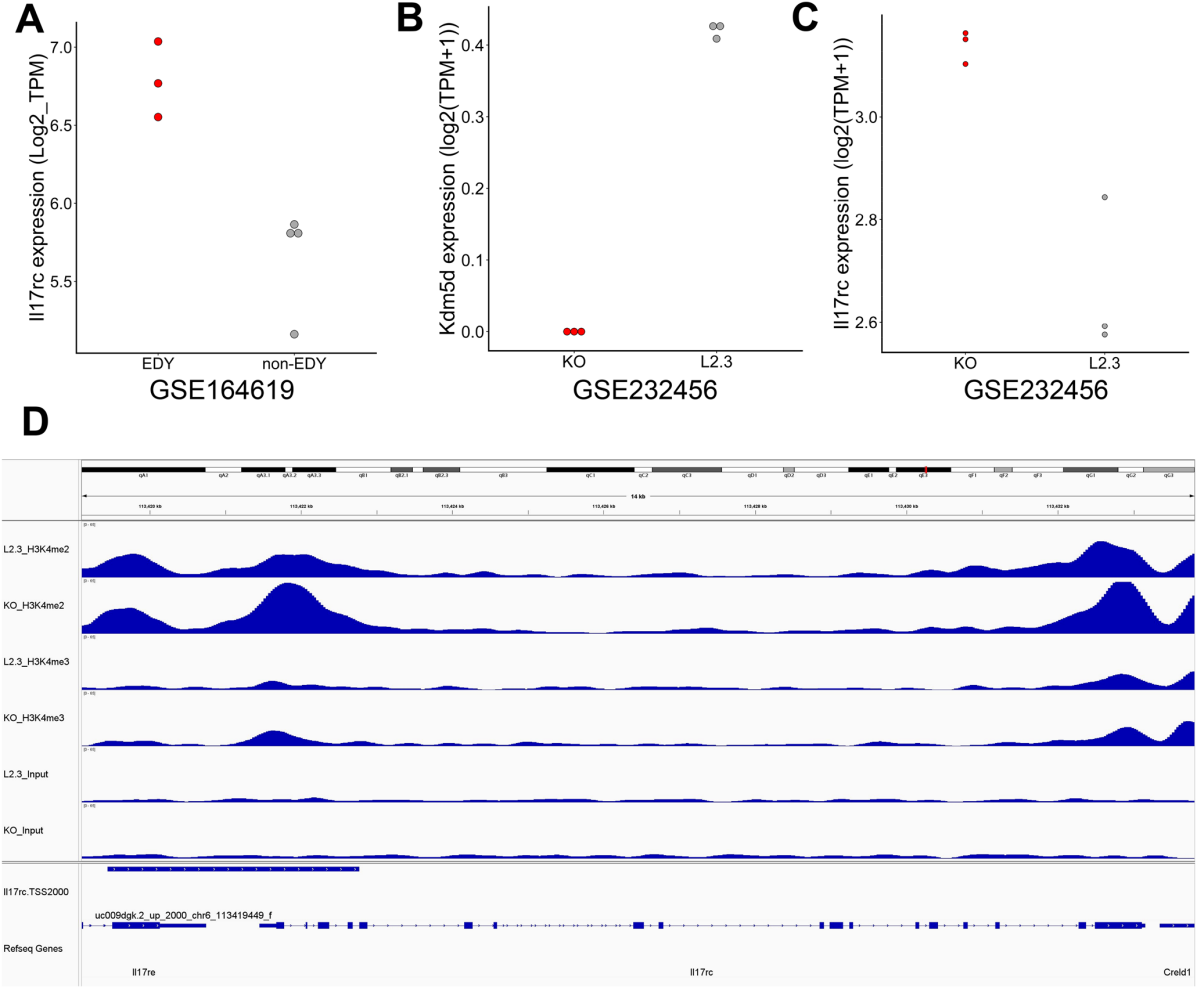
IL17RC expression and epigenetic regulation in epithelial cells with EDY. (A) Dot plot shows the expression of Il17rc between EDY and non‐EDY in the GSE164619 OPMD samples. (B) Dot plot shows the expression of Kdm5d between KO and L2.3 in GSE232456. (C) Dot plot shows the expression of Il17rc between KO and L2.3 in GSE232456. (D) ChIP‐seq analysis for H3K4me3 and H3K4me2 marks at the IL17RC gene locus. [Color figure can be viewed at wileyonlinelibrary.com]

The EDY‐related differences in oncogenic pathway activities may resemble an altered communication between epithelial cells and stromal cells in normal tissue and during malignant progression. In the past, several studies reported higher IL‐17 signaling in the malignant progression of oral premalignant lesions into OSCC [32, 33]. CellChat revealed distinct stromal‐epithelial interactions in leukoplakia, with IL17 signaling present only in EDY+ epithelial cells and absent in non‐EDY cells (Figure 4B). The analysis identified epithelial cells with EDY as targets, whereas endothelial cells, fibroblasts, macrophages, and T cells acted as senders (Figure 4B,C). This finding was further supported by a higher expression of IL17RC in EDY+ epithelial cells as compared to their non‐EDY counterparts (expression frequency: 28.6% vs. 16.9%, respectively) (Figure 4D). A positive association between EDY and IL17RC expression was also evident in potentially premalignant lesions of a chemically induced mouse model (*Z* = 2.23, *p* = 0.028; permutation test, Figure 5A). This suggests a potential link between IL17RC expression and EDY and underscores the potential role of EDY in facilitating an inflammatory microenvironment conducive to tumor progression into OSCC [34].

### 3.7 Epigenetic Regulation of IL17RC Expression by KDM5D

Given that EDY was associated with higher IL17RC in premalignant epithelial cells, the potential mode of action was further explored based on published key findings: (i) Recent reports indicate an epigenetic regulation of IL17RC gene expression [35, 36], and (ii) KDM5D, a tumor suppressive chromatin modifier encoded on the Y chromosome, demethylates di‐ and trimethyl H3K4, thereby repressing gene expression by epigenetic regulation [37, 38]. To determine whether KDM5D regulates IL17RC expression via histone modification, bulk RNA‐seq (GSE232456) and ChIP‐seq data (GSE232457) for a metastatic colorectal cancer cell line with or without Kdm5d knockout, which was established from a male mouse [39], were analyzed. Indeed, Il17rc expression was significantly increased by the Kdm5d knockout (*t*‐test, *p* = 0.006; Cohen's *d* = 4.32) accompanied by higher peaks for H3K4me2 and H3K4me3 at the Il17rc gene locus (Figure 5B–D). These data indicate an epigenetic regulation of IL17RC expression by KDM5D, which may also occur in oral epithelial cells.

### 3.8 Exploratory Analysis of EDY in Lung Precancerous Lesions

To explore whether EDY is also evident in premalignant lesions of other solid tumors, bulk RNA‐seq data (GSE49155) from a lung SCC study [40] with matched normal, premalignant and tumor samples from male patients were analyzed (GSE49155, Figure S6A). EDY was detected not only in three (P1‐3) out of four tumor samples (75%), but also in matched premalignant samples from two patients (P1‐2, 50%, Figure S6B). Only one patient (P4, 25%) showed no evidence of EDY in all samples tested. Although limited in sample size, this preliminary observation suggests that EDY can occur at early stages of lung tumorigenesis, supporting the broader relevance of EDY/LOY beyond OPMDs and warranting further investigation in further study.

## 4 Discussion

Head and neck cancer is a common malignancy with higher incidence and mortality in men than women [41]. Squamous cell carcinoma is the most prevalent type, accounting for over 90% of cases [42]. In men with HNSCC, LOY is associated with poor prognosis, whereas high expression of Y chromosome genes correlates with improved survival [10, 43, 44]. The difference in survival has been attributed to an enhanced immune response involving B and T cells, suggesting an association between Y chromosome gene expression and the immune phenotype in HNSCC [44]. However, the knowledge of LOY and EDY in potentially premalignant lesions and the malignant progression of OPMDs to OSCC is limited.

OPMD is a major health concern due to its high prevalence with a risk of malignant progression to OSCC. The risk of malignant progression from OPMD to OSCC varies widely from 3% to 50% [45]. Although many potential biomarkers have been proposed for progression, none are yet clinically applicable due to variability and lack of validation [46]. Autofluorescence imaging shows promise for assessing malignant progression in oral leukoplakia, but further large‐scale studies are needed [47]. More recently, machine learning has outperformed traditional methods in predicting OPMD risk, but the performance depends on the quality of the input data [48]. In conclusion, despite advances in experimental and clinical research, reliable prognostic markers to predict OPMD progression remain elusive. By studying Y chromosome gene expression using bulk RNA‐seq data from potentially premalignant lesions, valuable insights on the potential impact of EDY in the early steps of oral carcinogenesis were gained. Accordingly, this study is the first to demonstrate the presence of EDY in premalignant lesions of both patients and a pre‐clinical mouse model. In addition, EDY was also evident in oral epithelial cells from histologically normal tissue and clinical leukoplakia at the single‐cell level. These EDY+ epithelial cells exhibited a characteristic gene expression profile associated with a higher potential for malignant progression [13]. A recent study by Celli et al. [49] investigating the elimination of the Y chromosome using the CRISPR/Cas9 system in the male retinal epithelial cell line ARPE‐19 provides compelling experimental evidence that LOY increases cellular motility and invasiveness while simultaneously slowing proliferation. This supports the notion that epithelial cells with EDY resemble molecular features of carcinoma in situ and cancer.

Mechanistically, the data of this study suggest enhanced communication between EDY+ epithelial cells and various immune and stromal cells via IL17 signaling. Notably, the cell‐to‐cell communication via IL17‐IL17RA/C appears to be more prominent in OPMD with EDY+ epithelial cells, which is less evident in normal tissue or OSCC. This finding suggests a unique role of an inflammatory microenvironment in promoting malignant progression, which is supported by previous studies demonstrating an inflammatory milieu with higher IL17 levels in potentially premalignant lesions of the 4‐NQO mouse model and patients [32, 33]. It is worth noting that both studies reported a decrease in this inflammatory phenotype in HNSCC. The context‐dependent regulation and function of IL17 signaling may be due to the accumulation of additional genetic or epigenetic events during malignant progression. The elevated IL17‐IL17RC signaling could also explain the higher PI3K and JAK–STAT pathway activity in EDY+ epithelial cells in leukoplakia [50, 51, 52]. Both pathways have been shown to promote the malignant progression from premalignant lesions to OSCC in preclinical models [53, 54, 55, 56]. Finally, the potential mechanisms by which EDY promotes the cell‐to‐cell communication between epithelial cells and the inflammatory microenvironment via IL17‐IL17RC were explored in this study and indicated a key role for KDM5D. KDM5D is a Y chromosome encoded histone demethylase that removes H3K4me2/3 marks to repress gene expression [37, 38]. Accordingly, murine colon cancer cells with Kdm5d gene silencing demonstrated an increased Il17rc expression, likely due to the accumulation of H3K4me2/3 marks resulting from the loss of KDM5D's demethylase activity.

Although this study provides valuable and novel insights, it is not without limitations. First, the number of cases for this study with an exploratory study design was limited by the focus on males and the frequency of samples with EDY/LOY. The primary aim of this work was hypothesis‐generating—to explore whether EDY/LOY might serve as early male‐specific indicators of malignant progression in OPMD. Due to the limited sample size, conducting robust analyses by adjusting for additional prognostic variables was not feasible. The borderline *p*‐value observed in the small clinical cohort may reflect limited statistical power rather than absence of effect, which is not uncommon in exploratory studies. However, this limitation is mitigated by supporting evidence from several independent patient cohorts and pre‐clinical mouse models with either genomics and/or bulk RNA‐seq data, and a comprehensive analysis of scRNA‐seq data. Moreover, the absence of statistical significance in the clinical cohort does not contradict our hypothesis, in which EDY/LOY represents one of several, but not the only, male‐specific risk factors contributing to malignant transformation. However, further validation of the key findings and main conclusions regarding clinical relevance is needed using samples from larger cohorts and ultimately require a prospective study design. However, in the context of OPMD this is an ambiguous challenge and long‐term endeavor. Another limitation is the focus on transcript levels which may only partially reflect protein expression of Y chromosome genes. However, most protein‐based assays, including immunohistochemical staining, are inaccurate in reporting Y chromosome‐encoded protein expression due to high sequence homology with X chromosome‐encoded proteins [57, 58]. For example, KDM5C as KDM5D homolog on the X chromosome encodes a protein with approximately 84% sequence similarity to KDM5D [38]. Developing more specific antibodies or employing new proteomic methodology could help to solve these problems. Finally, the predicted model of how EDY and the inflammatory milieu promote malignant progression to OSCC is based on bioinformatic analysis, and a more detailed functional characterization has been initiated to substantiate key conclusions about causal interactions between EDY, IL17 signaling, PI3K and JAK–STAT pathway activity in vitro and in vivo preclinical models.

The discovery of EDY/LOY in early oral precancer stages has important clinical value. In practice, finding EDY/LOY‐positive cells could improve current diagnostic methods by helping clinicians to better assess risk and monitor patients more closely. Patients with EDY/LOY changes might need more careful follow‐up. From a research point of view, presented data support further studies into the role of the Y chromosome in cancer, which could lead to new ways of managing precancer and treating early‐stage cancers.

## 5 Conclusions and Perspectives

The findings indicate that EDY/LOY represents an early molecular event in male oral carcinogenesis, occurring before overt malignant transformation. This highlights the potential of EDY/LOY as a biomarker to improved risk stratification, enabling targeted surveillance or early intervention for patients with high‐risk lesions. By extending previous associations between LOY and poor prognosis in HNSCC, the results underscore the importance of Y chromosome gene expression in maintaining epithelial integrity and suppressing malignant progression. Emerging evidence from lung suggests that similar molecular alterations may arise early in tumorigenesis across various tissues, indicating a potentially generalizable role for EDY/LOY. Future joint studies using large, multi‐tissue datasets will facilitate comparisons across cancer types and improve the understanding of how EDY/LOY contributes to cancer development. To further substantiate this findings, bioinformatic analyses need to be complement with experimental validation. Functional approaches, including gene perturbation, cellular phenotyping, and organoid modeling, have to be employed to elucidate the biological consequences and mechanisms of EDY/LOY in epithelial transformation. In addition, some high‐risk OPMD types‐such as proliferative verrucous leukoplakia (PVL)‐have to be thoroughly investigated, which is currently limited by the lack of well‐annotated public datasets. Advancing the understanding in these areas will require studies based on dedicated and carefully characterized cohorts.

## Conflicts of Interest

The authors declare no conflicts of interest.

## Supporting information


**FIGURE S1:** Y chromosome‐related GSVA scores across different pathological groups of the 4‐NQO mouse model. Dot plots depict GSVA scores based on protein coding Y chromosome genes in tongue (normal), premalignant (OPMD) and tumor tissue from individual dataset: GSE75421 (A), GSE164619 (B), and GSE229289 (C).

**FIGURE S2:** Y chromosome‐related GSVA score and genomic Y chromosome signal in several patient cohorts. Dot plots illustrate the GSVA scores based on Y chromosome genes for samples from females and males in two datasets: GSE227919 (A) and GSE26549 (B). (C) Dot plots shows the Y chromosome signal and adjusted Y chromosome signal for progressor and non‐progressor samples from male samples in the Amsterdam UMC cohort. (D) Dot plot shows the adjusted Y chromosome signal for OPMD samples with or without progression into OSCC for males of the Amsterdam UMC cohort.

**Figure S3:** Analysis of EDY and quality control of scRNA‐seq data from GSE181919. (A) Bar plot shows the relative frequency of EDY in female and male samples from the GSE181919 dataset. Density plots show the distribution of mitochondrial gene transcripts (B) and ribosomal gene transcripts (C) in percentage, and nFeatures (D) and nCounts of RNA transcripts (E) in epithelial cells of male samples.

**Figure S4:** Expression of the gene sets related to malignant progression in epithelial cells based on the GSE181919 dataset. Bubble plots show the expression of individual genes of the 14‐gene set (A, C) and the 24‐gene set (B, D) in all epithelial cells (B, C) from normal tissue (NL), leukoplakia (LP) and cancer (CA) or in epithelial cells with or without EDY in different tissues (C, D). Violin plots show the score based on the 14‐gene set (E) and based on the 24‐gene set (F) for epithelial cells from cancers of males from the GSE181919 dataset. *p ≤ 0.05, **p ≤ 0.01, ***p ≤ 0.001 and ****p < 0.0001.

**Figure S5:** Oncogenic pathway activity in epithelial cells of the GSE181919 dataset. Heatmap shows differences in oncogenic pathway activity based on PROGENy scores for epithelial cells from normal tissue (NL), leukoplakia (LP) and cancer (CA) of males.

**Figure S6:** Workflow and analysis of the lung cohort (GSE49155). (A) Schematic illustration of the samples with available transcriptome data for males. Created in BioRender. Han, R. (2024) BioRender.com/a46d858 (B) Dot plot shows Y chromosome‐related GSVA scores for normal, premalignant and tumor samples for individual patients.

**Table S1:** List of the public data sets, organism and tissue sources used in this study.

**Table S2:** Summary of the software and packages used for the analysis.

## Data Availability

The data that support the findings of this study are available from the corresponding author upon reasonable request.
