# Supplementary material for: Loss of the Y Chromosome in Oral Potentially Premalignant Disorders Predicts Malignant Progression: An Integrative Cross‐Species Multi‐Cohort Bioinformatic Study
Source: Head Neck. 2025 Oct 22;48(3):782–93. doi: 10.1002/hed.70070 (PMC12891753; doi:10.1002/hed.70070)
Supplement: Supplementary file 1 — FIGURE S1: Y chromosome‐related GSVA scores across different pathological groups of the 4‐NQO mouse model. Dot plots depict GSVA scores based on protein coding Y chromosome genes in tongue (normal), premalignant (OPMD) and tumor tissue from individual dataset: GSE75421 (A), GSE164619 (B), and GSE229289 (C). [file HED-48-782-s005.pptx]

## Slide 1
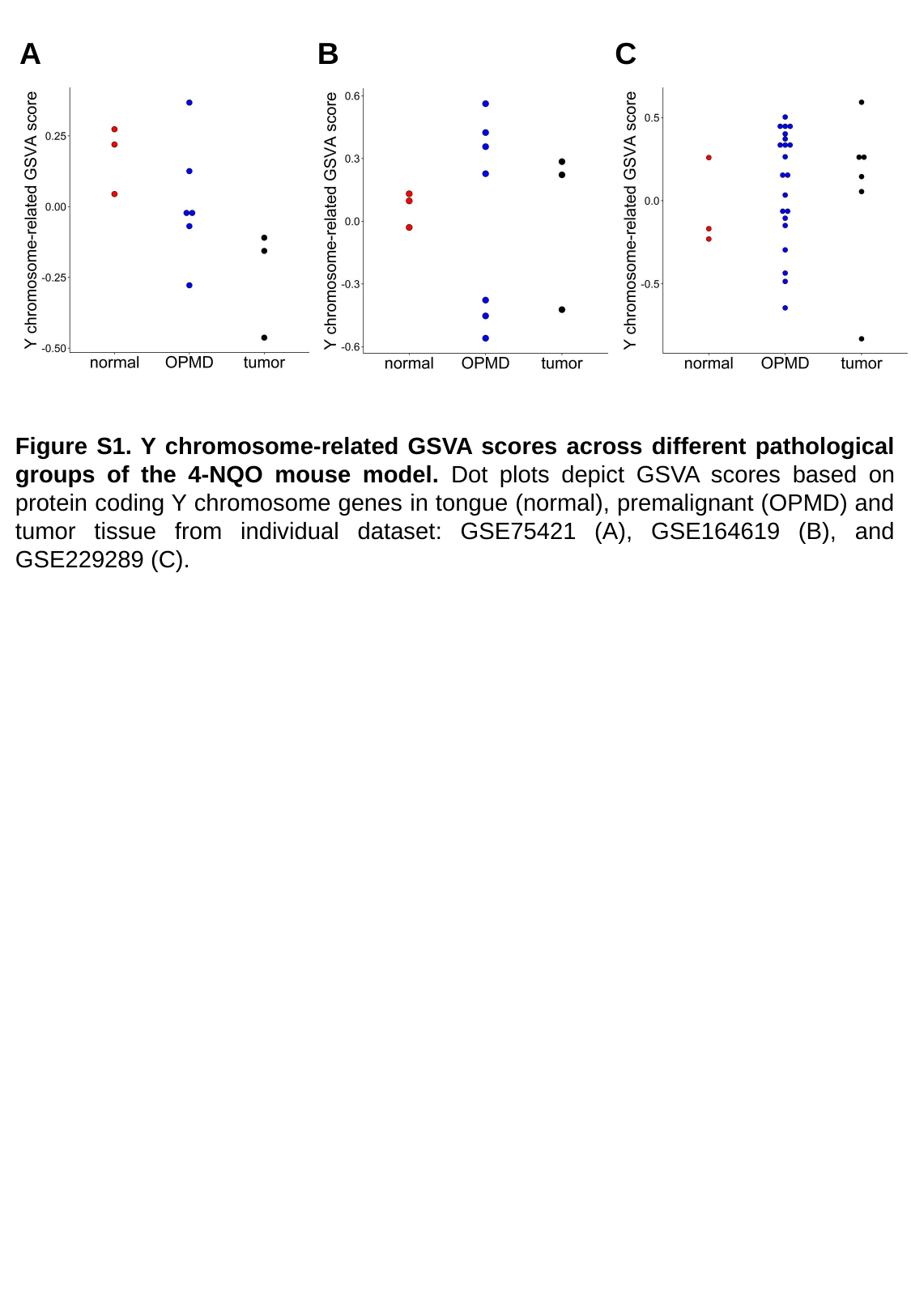

A
B
C
Figure S1. Y chromosome-related GSVA scores across different pathological groups of the 4-NQO mouse model. Dot plots depict GSVA scores based on protein coding Y chromosome genes in tongue (normal), premalignant (OPMD) and tumor tissue from individual dataset: GSE75421 (A), GSE164619 (B), and GSE229289 (C).
