# Supplementary material for: Loss of the Y Chromosome in Oral Potentially Premalignant Disorders Predicts Malignant Progression: An Integrative Cross‐Species Multi‐Cohort Bioinformatic Study
Source: Head Neck. 2025 Oct 22;48(3):782–93. doi: 10.1002/hed.70070 (PMC12891753; doi:10.1002/hed.70070)
Supplement: Supplementary file 2 — FIGURE S2: Y chromosome‐related GSVA score and genomic Y chromosome signal in several patient cohorts. Dot plots illustrate the GSVA scores based on Y chromosome genes for samples from females and males in two datasets: GSE227919 (A) and GSE26549 (B). (C) Dot plots shows the Y chromosome signal and adjusted Y chromosome signal for progressor and non‐progressor samples from male samples in the Amsterdam UMC cohort. (D) Dot plot shows the adjusted Y chromosome signal for OPMD samples with or without progression into OSCC for males of the Amsterdam UMC cohort. [file HED-48-782-s004.pptx]

## Slide 1
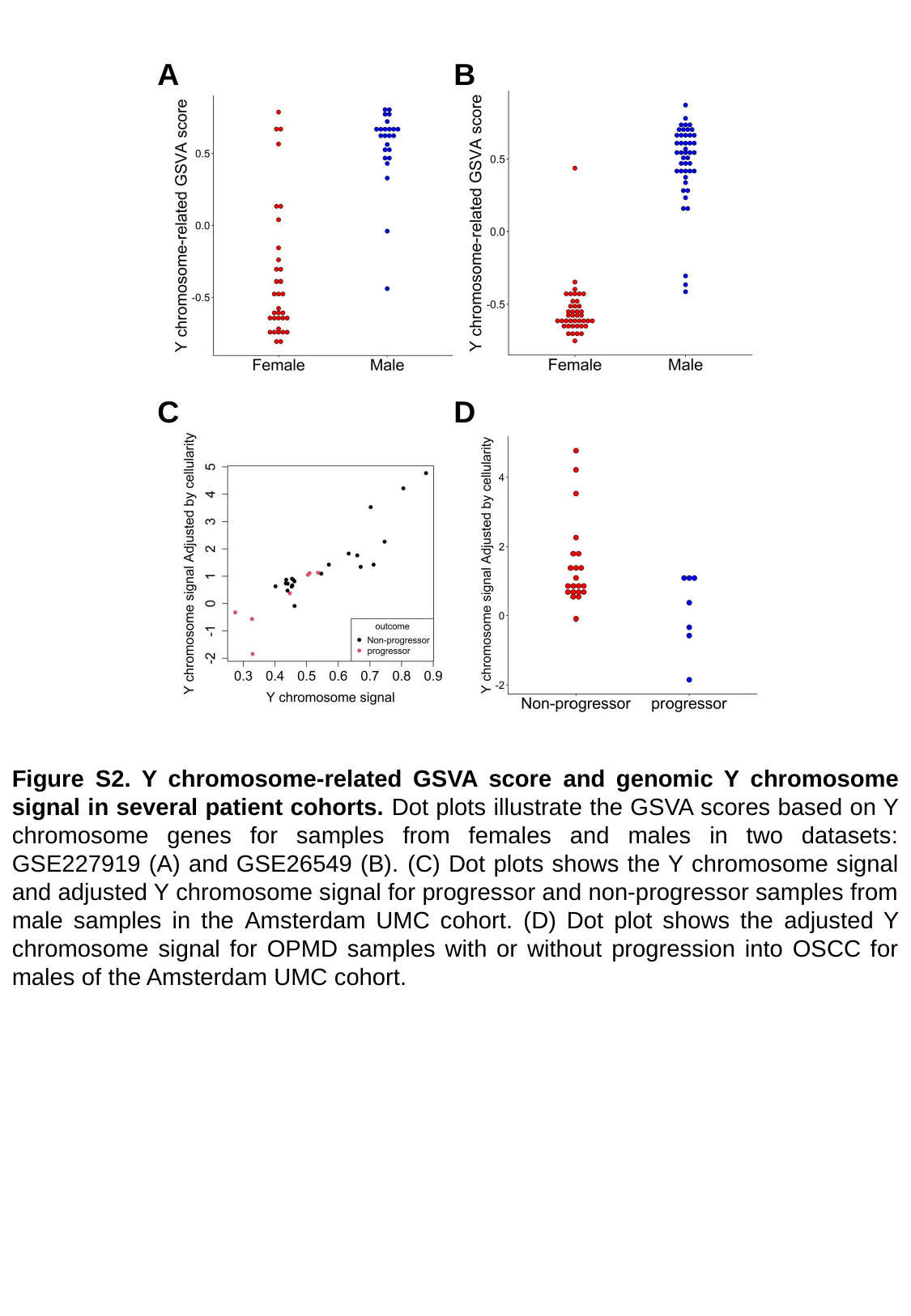

A
B
C
D
Figure S2. Y chromosome-related GSVA score and genomic Y chromosome signal in several patient cohorts. Dot plots illustrate the GSVA scores based on Y chromosome genes for samples from females and males in two datasets: GSE227919 (A) and GSE26549 (B). (C) Dot plots shows the Y chromosome signal and adjusted Y chromosome signal for progressor and non-progressor samples from male samples in the Amsterdam UMC cohort. (D) Dot plot shows the adjusted Y chromosome signal for OPMD samples with or without progression into OSCC for males of the Amsterdam UMC cohort.
