# Supplementary material for: Loss of the Y Chromosome in Oral Potentially Premalignant Disorders Predicts Malignant Progression: An Integrative Cross‐Species Multi‐Cohort Bioinformatic Study
Source: Head Neck. 2025 Oct 22;48(3):782–93. doi: 10.1002/hed.70070 (PMC12891753; doi:10.1002/hed.70070)
Supplement: Supplementary file 3 — Figure S3: Analysis of EDY and quality control of scRNA‐seq data from GSE181919. (A) Bar plot shows the relative frequency of EDY in female and male samples from the GSE181919 dataset. Density plots show the distribution of mitochondrial gene transcripts (B) and ribosomal gene transcripts (C) in percentage, and nFeatures (D) and nCounts of RNA transcripts (E) in epithelial cells of male samples. [file HED-48-782-s001.pptx]

## Slide 1
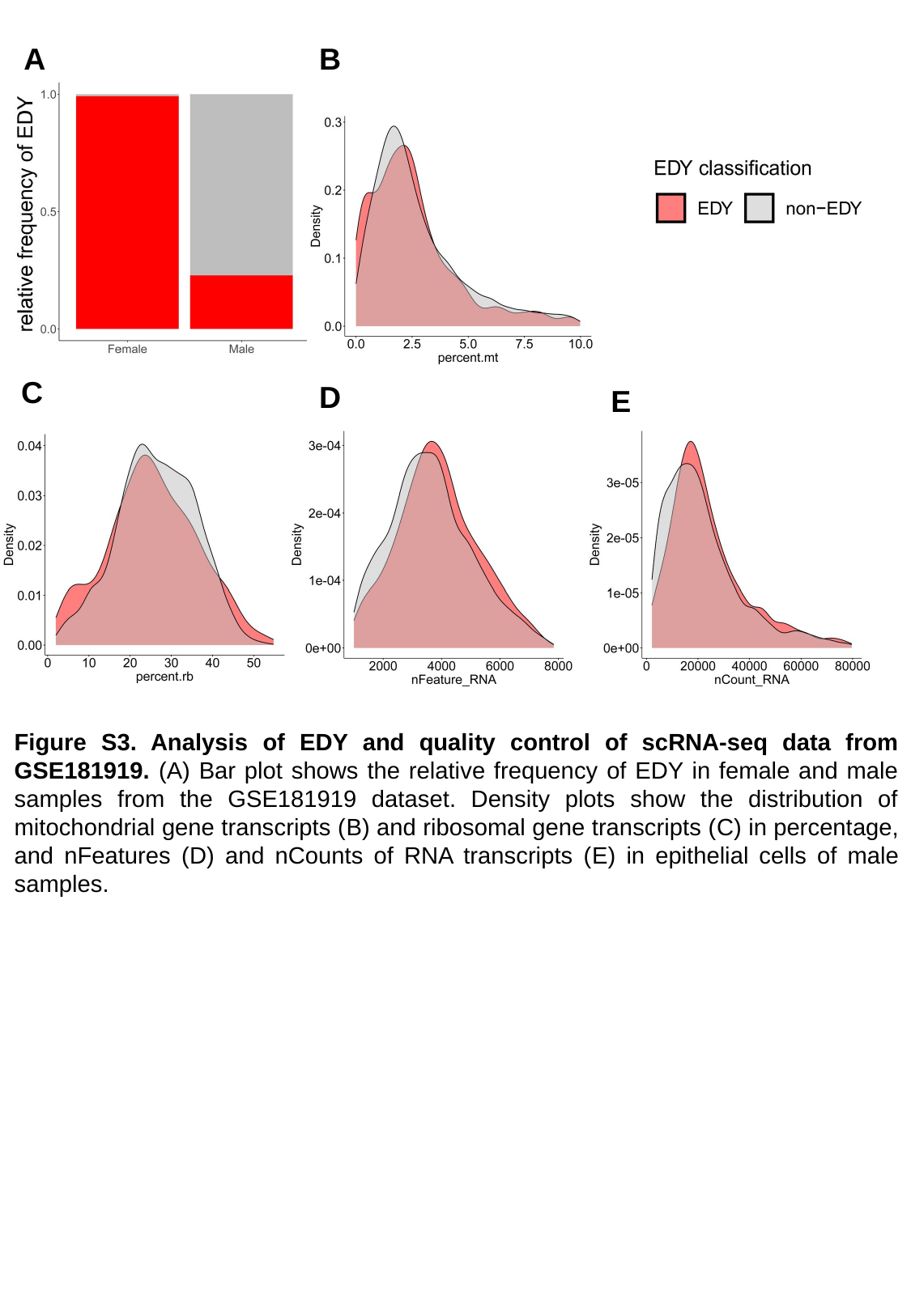

A
B
C
D
E
Figure S3. Analysis of EDY and quality control of scRNA-seq data from GSE181919. (A) Bar plot shows the relative frequency of EDY in female and male samples from the GSE181919 dataset. Density plots show the distribution of mitochondrial gene transcripts (B) and ribosomal gene transcripts (C) in percentage, and nFeatures (D) and nCounts of RNA transcripts (E) in epithelial cells of male samples.
