# Supplementary material for: Loss of the Y Chromosome in Oral Potentially Premalignant Disorders Predicts Malignant Progression: An Integrative Cross‐Species Multi‐Cohort Bioinformatic Study
Source: Head Neck. 2025 Oct 22;48(3):782–93. doi: 10.1002/hed.70070 (PMC12891753; doi:10.1002/hed.70070)
Supplement: Supplementary file 4 — Figure S4: Expression of the gene sets related to malignant progression in epithelial cells based on the GSE181919 dataset. Bubble plots show the expression of individual genes of the 14‐gene set (A, C) and the 24‐gene set (B, D) in all epithelial cells (B, C) from normal tissue (NL), leukoplakia (LP) and cancer (CA) or in epithelial cells with or without EDY in different tissues (C, D). Violin plots show the score based on the 14‐gene set (E) and based on the 24‐gene set (F) for epithelial cells from cancers of males from the GSE181919 dataset. *p ≤ 0.05, **p ≤ 0.01, ***p ≤ 0.001 and ****p < 0.0001. [file HED-48-782-s006.pptx]

## Slide 1
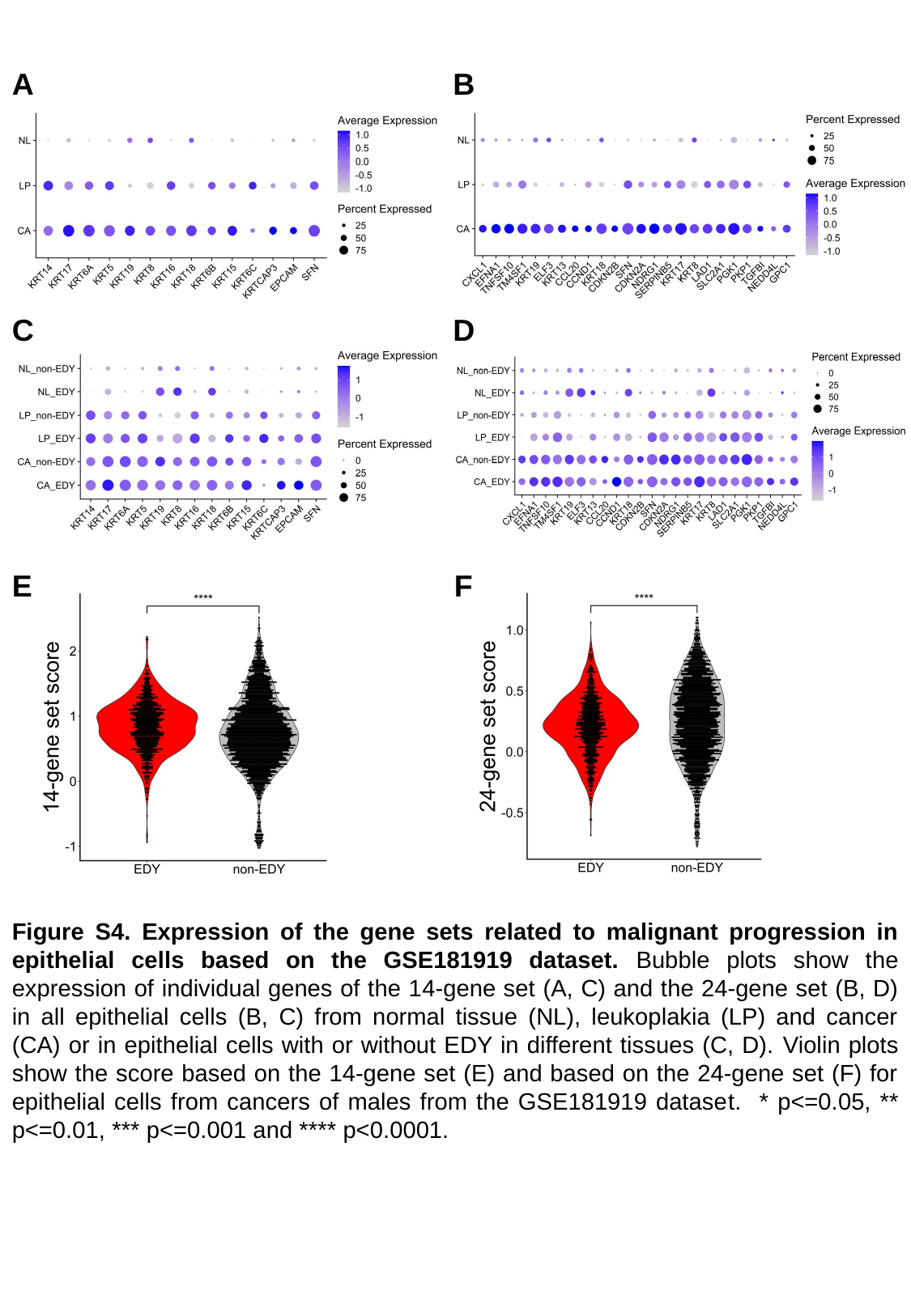

A
B
C
D
E
F
Figure S4. Expression of the gene sets related to malignant progression in epithelial cells based on the GSE181919 dataset. Bubble plots show the expression of individual genes of the 14-gene set (A, C) and the 24-gene set (B, D) in all epithelial cells (B, C) from normal tissue (NL), leukoplakia (LP) and cancer (CA) or in epithelial cells with or without EDY in different tissues (C, D). Violin plots show the score based on the 14-gene set (E) and based on the 24-gene set (F) for epithelial cells from cancers of males from the GSE181919 dataset. * p<=0.05, ** p<=0.01, *** p<=0.001 and **** p<0.0001.
