# Supplementary material for: Loss of the Y Chromosome in Oral Potentially Premalignant Disorders Predicts Malignant Progression: An Integrative Cross‐Species Multi‐Cohort Bioinformatic Study
Source: Head Neck. 2025 Oct 22;48(3):782–93. doi: 10.1002/hed.70070 (PMC12891753; doi:10.1002/hed.70070)
Supplement: Supplementary file 5 — Figure S5: Oncogenic pathway activity in epithelial cells of the GSE181919 dataset. Heatmap shows differences in oncogenic pathway activity based on PROGENy scores for epithelial cells from normal tissue (NL), leukoplakia (LP) and cancer (CA) of males. [file HED-48-782-s003.pptx]

## Slide 1
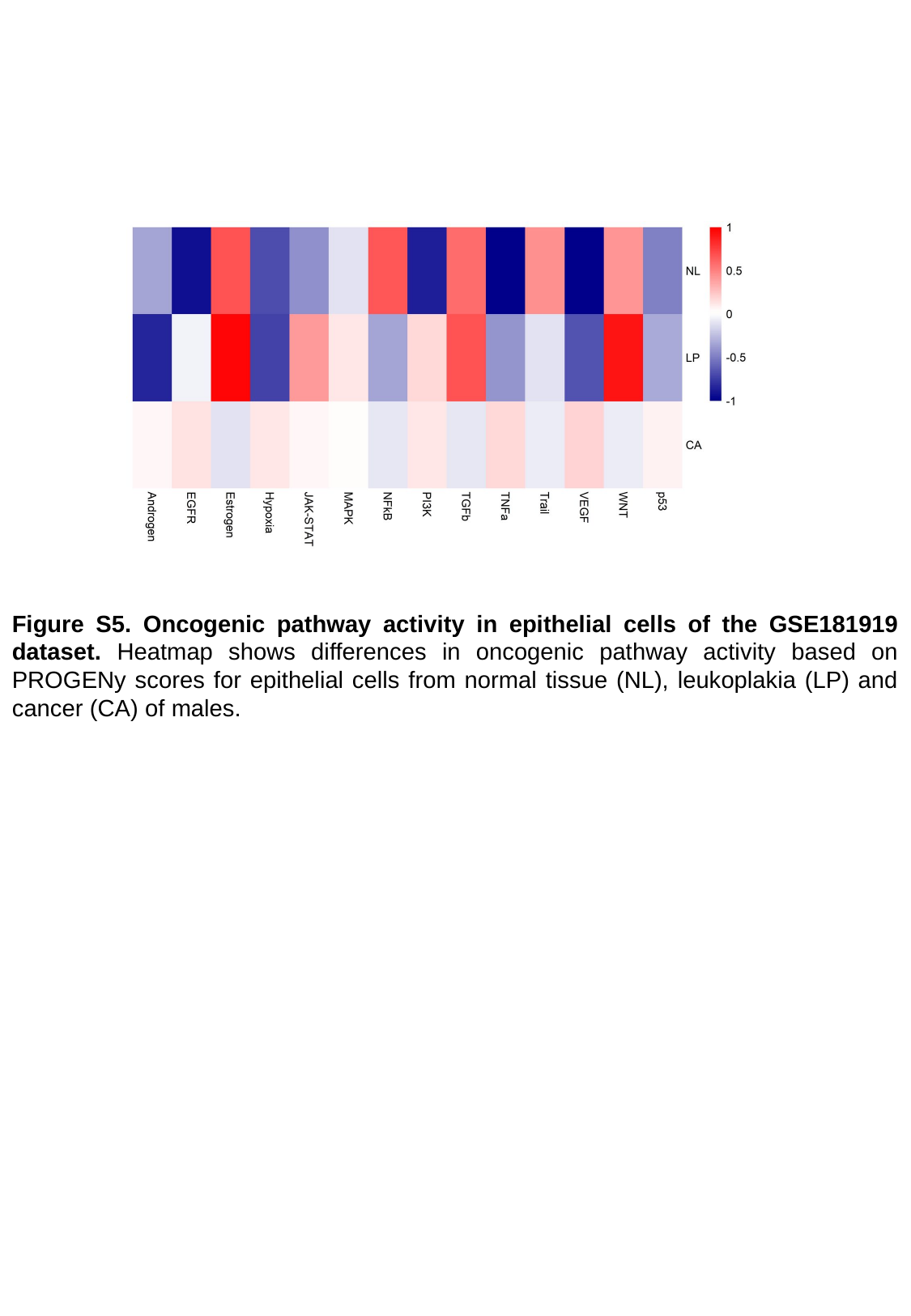

Figure S5. Oncogenic pathway activity in epithelial cells of the GSE181919 dataset. Heatmap shows differences in oncogenic pathway activity based on PROGENy scores for epithelial cells from normal tissue (NL), leukoplakia (LP) and cancer (CA) of males.
