# Supplementary material for: Loss of the Y Chromosome in Oral Potentially Premalignant Disorders Predicts Malignant Progression: An Integrative Cross‐Species Multi‐Cohort Bioinformatic Study
Source: Head Neck. 2025 Oct 22;48(3):782–93. doi: 10.1002/hed.70070 (PMC12891753; doi:10.1002/hed.70070)
Supplement: Supplementary file 6 — Figure S6: Workflow and analysis of the lung cohort (GSE49155). (A) Schematic illustration of the samples with available transcriptome data for males. Created in BioRender. Han, R. (2024) BioRender.com/a46d858 (B) Dot plot shows Y chromosome‐related GSVA scores for normal, premalignant and tumor samples for individual patients. [file HED-48-782-s007.pptx]

## Slide 1
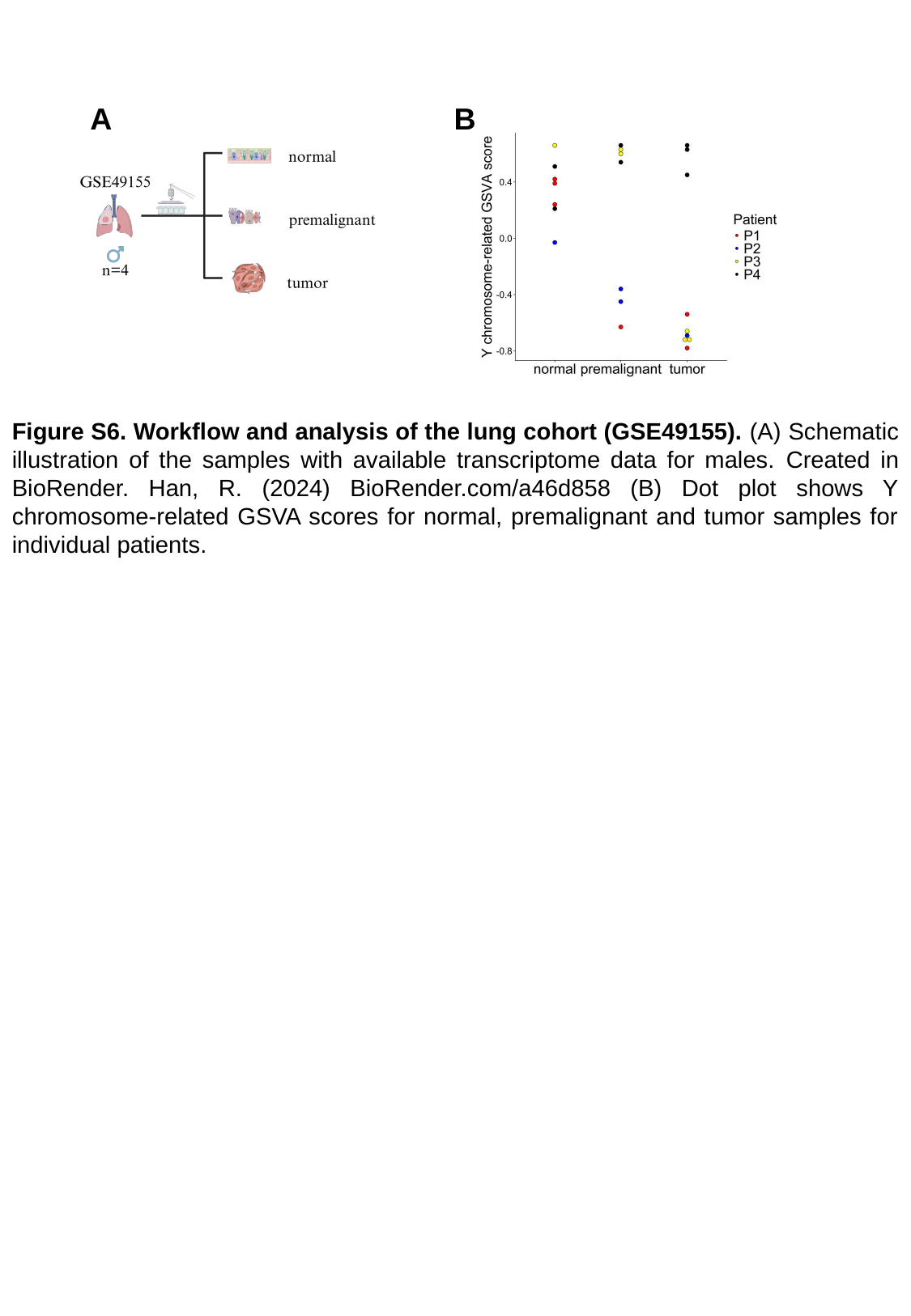

A
B
Figure S6. Workflow and analysis of the lung cohort (GSE49155). (A) Schematic illustration of the samples with available transcriptome data for males. Created in BioRender. Han, R. (2024) BioRender.com/a46d858 (B) Dot plot shows Y chromosome-related GSVA scores for normal, premalignant and tumor samples for individual patients.
